# Gene-environment dependencies lead to collider bias in models with polygenic scores

**DOI:** 10.1038/s41598-021-89020-x

**Published:** 2021-05-04

**Authors:** Evelina T. Akimova, Richard Breen, David M. Brazel, Melinda C. Mills

**Affiliations:** 1 Department of Sociology, University of Oxford, Oxford, OX1 1JD UK; 2 Leverhulme Centre for Demographic Science, University of Oxford, Oxford, OX1 1JD UK; 3 Nuffield College, University of Oxford, Oxford, OX1 1NF UK

**Keywords:** Genetics, Behavioural genetics, Genetic markers, Population genetics, Quantitative trait

## Abstract

The application of polygenic scores has transformed our ability to investigate whether and how genetic and environmental factors jointly contribute to the variation of complex traits. Modelling the complex interplay between genes and environment, however, raises serious methodological challenges. Here we illustrate the largely unrecognised impact of gene-environment dependencies on the identification of the effects of genes and their variation across environments. We show that controlling for heritable covariates in regression models that include polygenic scores as independent variables introduces endogenous selection bias when one or more of these covariates depends on unmeasured factors that also affect the outcome. This results in the problem of conditioning on a collider, which in turn leads to spurious associations and effect sizes. Using graphical and simulation methods we demonstrate that the degree of bias depends on the strength of the gene-covariate correlation and of hidden heterogeneity linking covariates with outcomes, regardless of whether the main analytic focus is mediation, confounding, or gene × covariate (commonly gene × environment) interactions. We offer potential solutions, highlighting the importance of causal inference. We also urge further caution when fitting and interpreting models with polygenic scores and non-exogenous environments or phenotypes and demonstrate how spurious associations are likely to arise, advancing our understanding of such results.

## Introduction

The importance of understanding the joint contributions of genetic and environmental variation underlying complex traits is widely recognised. The rise of polygenic scores has resulted in a surge of studies investigating the mediating and moderating roles of environments along with genetic confounding^1^ (i.e., whether genes confound associations between environments or phenotypes). Yet, disentangling the relative importance of polygenic scores and environmental covariates is difficult. Various methodological concerns have been raised, including but not limited to, the power and predictive accuracy of polygenic scores^2–4^ and the non-exogenous nature of environmental exposures and their consequences^5–7^. Moreover, genes and environments do not operate independently, necessitating greater scrutiny of both conventional models and new methods addressing gene-environment-trait correlations^8,9^. Here we address further methodological problems arising from gene-environment correlations that have gone largely unrecognised yet make identification of causal effects and the accurate estimation of associations even more challenging.

We illustrate how the presence of genetic predispositions associated with exposures to an environment (or phenotype in cases of genetic confounding) introduces *endogenous selection bias* in a regression analysis. Heritable covariates in regression models with polygenic scores are endogenous variables, and this can give rise to the problem of conditioning on a collider. Collider bias is an important statistical problem that destabilises regression models and it can arise for a variety of reasons, including sample selection and attrition^10^. We demonstrate that collider bias is likely to occur not only in genetic association studies but also in other analyses where polygenic scores are included, regardless of whether the main focus is mediation, confounding, or gene × environment (G × E) interaction.

Moreover, the issue we describe here is linked to a growing body of literature showing the heritability of environments, known as gene-environment correlation (rGE) and pleiotropy. To date, discussion of the methodological implications of these findings has focussed on the implications for G × E interaction studies (e.g. Dudbridge and Fletcher^7^) and studies using polygenic scores as instrumental variables (e.g. Conley and Zhang^11^). We expand this focus and show that if both genetic and environmental covariates are included in a statistical model, gene-environment correlations may lead to spurious estimates and effect sizes. Understanding the mechanisms that generate these dependencies is crucial for how we may solve the issue. We emphasise that researchers must grasp the conceptual differences between passive, evocative, and active gene-environment correlations^12^ and the potential sources of endogeneity of environmental covariates in order to obtain results that are not biased due to conditioning on a collider.

We use a graphical approach to demonstrate these methodological problems, illustrated by simulations. We then discuss the consequences of the bias in linear models and offer some potential solutions. The problems outlined here are relevant for making both causal and non-causal claims, with serious implications for the interpretation of results.

## Endogenous selection bias

The notion of *endogenous selection bias* arises from the broader concept of *selection bias*. While the term *selection bias* is very widely used^13^, *endogenous selection bias* commonly arises in analyses in which we adjust for an endogenous variable—that is, a variable caused by other, unmeasured variables which also affect the outcome. In this case, bias arises through the adjusting variable operating as a collider. Collider bias was demonstrated by Day et al.^14^ in the context of genetic association studies where such biases led to false-positive and biologically spurious associations. Their investigation considered the case of sex and autosomal genetic variants associated with height: neither factor is plausibly correlated with the other but both have an effect on height. Day et al*.* showed that the inclusion of height as a covariate created a robust but biologically spurious association between sex and height-associated variants. The bias arose because the respondent's height is a collider variable—a direct product of another covariate (SNPs of height) and an outcome (sex).

In what follows we consider a situation in which genes and environment are correlated (for reasons discussed below) and the environmental variable(s) is affected by variables not measured in the study but which also affect the outcome. We then discuss the consequences of the resulting collider bias for both additive and G × E interaction models.

### Additive models with polygenic scores

The first type of model we consider is the rather straightforward design where polygenic scores and environmental covariates (or phenotypes if they are used as covariates) are jointly included as a set of predictors for an outcome of interest. Such models are intended to reveal whether genetic influences confound associations between environments and outcomes or whether environments are mediators of the link between genetic variants and phenotypes. Examples include linking health disparities with socio-economic outcomes such as the relationship between attention-deficit hyperactivity disorder (ADHD), its polygenic prediction, and IQ on educational outcomes among teenagers (e.g. Stergiakouli et al.^15^). Other examples include studies that examine genetic risk and lifestyle in relation to stroke and cardiovascular mortality (e.g. Rutten-Jacobs et al.^16^; Yun et al.^17^). Another example is where income and labour market outcomes are predicted by educational measures (e.g., grades, years of education) along with an educational attainment polygenic score (e.g. Ayorech et al.^18^; Papageorge and Thom^19^). Or the study of the variation of exam scores in relation to school types and polygenic prediction of education (e.g. Smith-Woolley et al.^20^).

All of these studies are similar with respect to the nature of environmental variables—they are not exogenous, being the direct or indirect products of polygenic scores which are also included into the models. Dependence of these covariates could arise through the inclusion of a phenotypic variable—the scenario that is prevalent in genetic confounding studies. For example, the ADHD polygenic score is directly linked to the diagnosis of ADHD, so once genetic risk and the corresponding phenotype are covariates, their dependency is present in the model.

Dependencies could also arise when gene-environment correlation (rGE) plays a role. rGE occurs when exposure to an environment depends on heritable inclinations^21^. While strictly speaking rGE is a statistical correlation between genetic variation and exposure to an environment, behavioral genetics theory distinguishes between passive, evocative, and active mechanisms^12,22^. Passive rGE arises because non-transmitted parental alleles may influence the rearing environment, which induces a correlation if parental characteristics are not controlled for. Moreover, Kong et al.^23^ demonstrate that the signals obtained from GWAS are likely to reflect both direct and genetic nurturing effects, which further contributes to our expectation of interdependency between polygenic scores and environments. Associations could also arise due to active and evocative selection in environments. Applying the polygenic prediction of educational attainment as an example, we see that it contributes to the variation of school grades^20^ which likely reflects active rGE (i.e., children selecting their environments for genetically influenced reasons). It could also be linked to school type since parents adjust their educational choices for children depending on their child’s characteristics which are partially due to genetic differences, reflecting evocative rGE (i.e., the child indirectly shapes the environment via the reaction of parent’s to the child’s behaviour)^24,25^.

It is also important to consider the role of pleiotropy as a cause of gene-environment dependencies. In general, pleiotropy refers to situations when one gene influences multiple traits or two traits share genetic variants. There are different mechanisms involved described by Van Rheenen et al.^26^ into horizontal, vertical, and spurious pleiotropy. Horizontal pleiotropy occurs when genetic variants are either linked to multiple traits, either directly or through a series of intermediate traits; vertical pleiotropy arises if we expect causation among a set of traits; and, spurious pleiotropy is the result of linkage disequilibrium (LD), misclassification, or other biases. The most relevant aspect for our argument is that any type of pleiotropy could cause gene-environment dependency. As noted in the literature^11,27^, pleiotropy is not unusual among heritable traits. Rather, the null hypothesis is that pleiotropy exists unless proven otherwise.

One way to assess whether consistent pleiotropy, at the level of observed genetic variance, exists is via genetic correlations (*r*_*g*_)^26,27^. To illustrate this point, we can consider studies on cardiovascular mortality and incidents of stroke which investigate the joint importance of polygenic risk scores and lifestyles^16,17^. Conceptualisation of lifestyle includes, but is not limited to, smoking and BMI, which both have moderate genetic correlations with heart attacks (*r*_*g*_ = 0.33 between heart attacks and smoking; *r*_*g*_ = 0.36 between heart attacks and BMI^28^). Therefore, we expect non-exogenous environments to vary depending on the values of polygenic scores. Such dependencies, when accompanied by hidden heterogeneity that links environments with outcomes, will result in endogenous selection bias, which we describe now.

**Figure 1:**
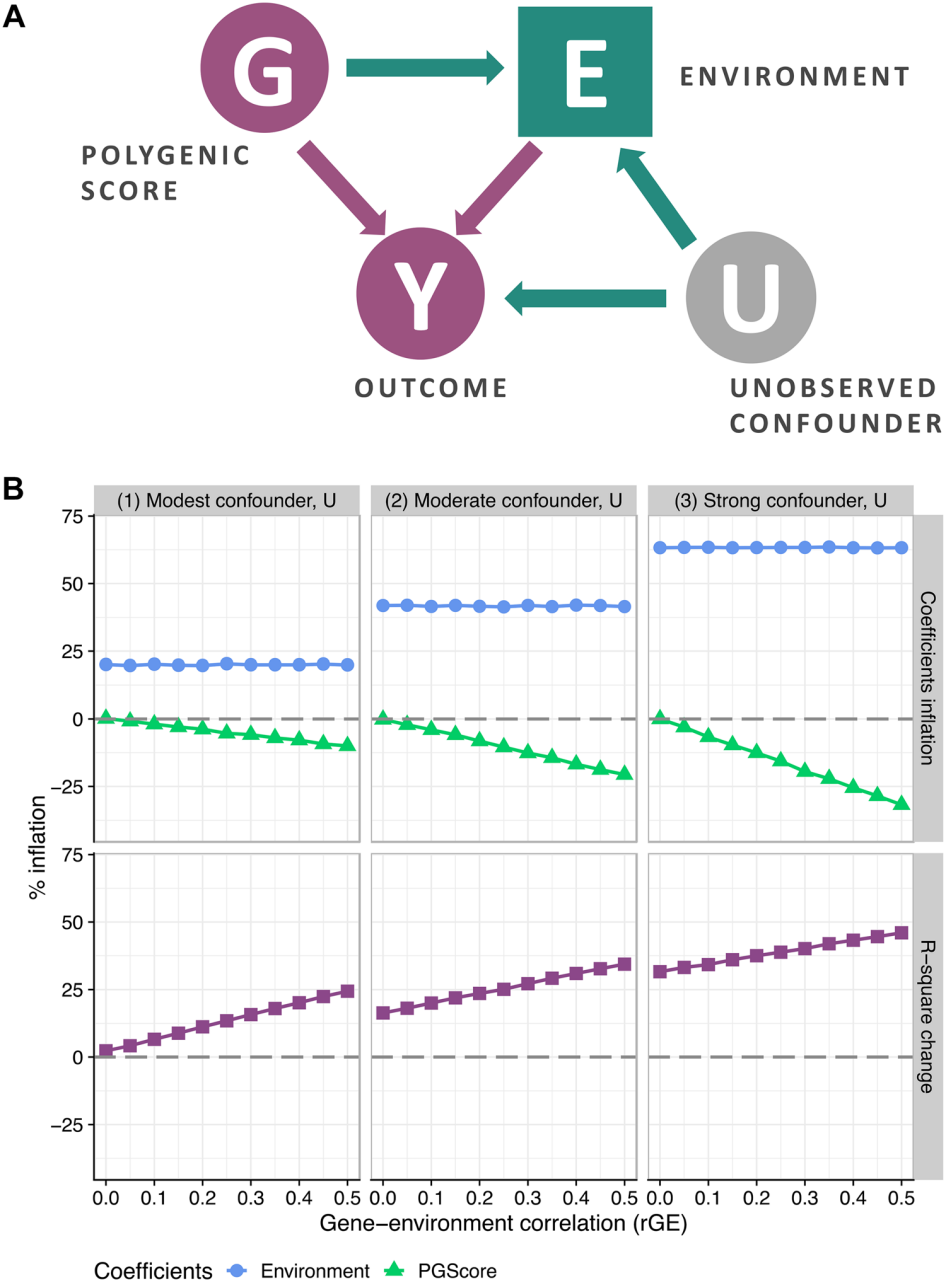
Collider bias in polygenic gene-environment models. Panel A. Schematic diagram of the collider bias which occurs between polygenic score, environment, and outcome in cases of gene-environment interdependence. Dark purple circles represent variables, unobserved confounders are shown in grey circles, collider variables are indicated by squares. By adding E into the model with the polygenic score G, we make E a collider. A collider that is not conditioned on, blocks the path between its sources (G and U); once a collider is controlled for, the path is opened as indicated by green nodes. Panel B (top). Spurious regression estimates for polygenic score and environment from the series of OLS simulations reflecting the range of gene-environment interdependence and the presence of modest, moderate, or strong confounder, U. Collider bias due to positive values of gene-environment correlation and the presence of uncontrolled confounder, which is positively correlated with covariate and outcome, results in deflation of polygenic score estimates. The degree of bias depends on the strength of unobserved confounder, U, and gene-covariate interdependence. Estimates of the environmental effect are upwardly biased but are not affected by the gene-environment correlation. Panel B (bottom). R-squared inflation plot from the series of OLS simulations; collider bias results in inflated values of explained variance statistics. R-squared statistics for the model with endogenous covariate and polygenic score includes not only the true share of the variance in Y explained by G and E (baseline estimate indicated by 0), but also the elements of variance that are due to gene-environment correlation and confounder(s), U.

Whether the aim is to address genetic confounding or to reveal mediation, models of this kind include polygenic scores and environments as predictors of an outcome of interest. The simple case is illustrated in Fig. 1A. The polygenic score, G, has an independent association with the outcome, Y, along with an indirect path through the environment, E. The exclusion from the model of the environmental covariate, E, results in the estimation of the total effect of G on Y, while the exclusion of G and the inclusion of E produces the association between E and Y, confounded by unobserved G.

The challenges for the model in Fig. 1A are to produce reliable estimates of the direct effects from G to Y and from E to Y in the face of confounding by the unmeasured U. Since the focus of our paper is not related to the issues of polygenic prediction per se, we do not include a discussion on the sources of bias between G and Y caused by confounders that are likely to arise due to assortative mating or population stratification, which have been amply explored elsewhere^29^. Here, we focus on the role of confounders of the link between environment and outcome.

The most important problem arising from the presence of unmeasured factors causing E and Y is that of non-exogenous environments. Factors such as socio-economic conditions, parental characteristics, health policies, cultural norms, and neighbourhood characteristics could cause E and Y to be correlated and, unless included in the model, they will be jointly present in the error structure of both variables. The issue is further problematic because the confounding can be driven by both observed and unobserved factors. Hence, even an extensive set of controls would not necessarily yield unbiased estimates if substantial confounding on unobservables remained unaddressed.

Moreover, unobserved confounder(s), U, linking E and Y biases not only their association but also the estimate of the direct effect of G on Y. This is driven by the fact that E is now a collider since it is a product of both G and U, as indicated by the arrows from U to E and G in Fig. 1A. It is known that if we do not control for a collider variable the path between its sources is blocked; however, once a collider is included in the set of covariates, the associated path is now open^30^. Accordingly, conditioning on E introduces a new path from G to E to Y through U: this is the green path denoted in Fig. 1A. This path is the source of the collider bias in these models.

We could simply omit the collider, E, from the model in Fig. 1A and estimate the total effect of G on Y, including the direct path from G to Y and the indirect path through E. However, while this might be desirable in some cases, models of this type usually aim to disentangle these paths leading to the necessity of including such covariates into the models along with polygenic scores. Here, it is important to clarify that the heritable covariate, E, is not a confounding variable because of the direction of the arrow, and thus we cannot treat it as an omitted variable that we wish to control for. It is rather a mechanism we condition on that introduces collider bias and requires other sets of solutions we discuss later in the paper.

To further illustrate this bias, we conducted a series of simulations of the simple linear model from Fig. 1A. We considered the presence of direct effects from G to Y and E to Y, allowed the G–E correlation to vary from 0 indicating no heritability to 0.5 indicating a highly heritable covariate, E, and included an uncontrolled confounder, U, which is positively correlated with both E and Y at a fixed value. We considered three scenarios where U is a modest, moderate, or strong confounder of E–Y association. The top panel of Fig. 1B illustrates the deviations of coefficients from the true simulated values. Notably, the presence of G–E correlation and an omitted confounder, U, where both U–E and U–Y associations are positive, results in the deflation of polygenic score estimates and inflation of environmental coefficients. Deflation of the G–Y association is greater with higher values of the G–E correlation, while the models without this association produced results free of collider bias. Moreover, the degree of bias also depends on the strength of the unobserved confounder, U. The path coefficient from E to Y is biased regardless of the strength of the G-E correlation reflecting the role of the omitted confounder, U, as a source of confounding of this path.

We also develop a mathematical expression of the bias for linear models, which we provide in the Supplementary Information. However, it is important to note that even though we consider simple linear models in our simulations and derivations, the bias would also arise in other types of regressions, e.g., logistic regression or Cox models. Since the graphical approach we follow does not require any parametric assumptions, the functional form describing the relationship between variables is not relevant.

It is also possible to exemplify the nature of inflation of the G-Y association by considering examples from the existing literature. Using the example of studies on cardiovascular mortality^17^ and incidents of stroke^16^ mentioned previously, the focus was to model the risk of strokes or cardiovascular mortality (Y) according to genetic risks (G) and lifestyle profiles (E), employing Cox proportional hazards models. A covariate such as a lifestyle may not be exogenous—individuals choose to follow healthy or less favourable behaviours based on various observed and unobserved factors. Hence, we expect not only G-E dependency, at least due to pleiotropy, but also the presence of unobserved confounders, U. Here, potential factors for U include, but are not limited to, childhood poverty, social or geographically-related deprivation, and socio-economic position^31–33^. This makes the estimation of G–Y association subject to collider bias since we introduced the path that goes through this set of unobserved confounders, U, into the statistical model. While one of the desires of these studies is to show that a healthy lifestyle potentially attenuates genetic risks, observed attenuations should be treated with caution.

Another instance is the study from Papageorge and Thom^19^, which we can analyse in a more straightforward manner because of the properties of linear models. Here the researchers regressed a polygenic score of educational attainment (G) on earnings (Y) and found that greater values of the genetic score were associated with greater income. What is relevant to our argument is that after the inclusion of educational controls (E) into the model, we observe a 59.5% decrease of the polygenic score coefficient (Table 5 Panel A in Papageorge and Thom^19^). In addition, the authors regress the educational attainment polygenic score (G) and years of schooling (E) on standardised job tasks (Y) to explain the trends they found in the models of earnings. If we take models on nonroutine analytic and nonroutine interactive tasks (where the association between polygenic score and outcome is also positive), we also see that the inclusion of educational controls results in about a 70% reduction in polygenic score coefficients. It is likely that such a change of G-Y association in both analyses is largely attributed to the extended set of educational controls, which includes both parental and respondent educational attainment. However, respondent’s own education in these models is an endogenous variable; hence, the decrease of polygenic score coefficients is likely to be due to collider bias at least to some extent. If we consider a moderate strength of association between the genetic score and respondent’s years of schooling along with additional assumptions about unobserved confounders linking educational attainment and the type of job tasks, we can show that around 15–20% of the polygenic score coefficient decrease is plausibly due to collider bias, following the derivations we include in the Supplementary Information.

In particular, if we consider the model with nonroutine interactive job tasks as the dependent variable (Table 6 in Papageorge and Thom^19^), we see that the baseline coefficient of the educational attainment polygenic score is 0.185. This reflects a model without any environmental and phenotypic covariates. In the model with educational controls, which are respondent’s years of schooling and parental education, the polygenic score coefficient drops to 0.055. Since the dependent variable is standardised, we can assess the relative importance of collider bias under additional assumptions. We assume that the coefficient of the correlation between polygenic score and respondent’s years of schooling is 0.300, and that there is an unobserved confounder U, which is positively correlated with both years of schooling and job task with coefficients of 0.250 (for example, living in advantaged higher socio-economic neighbourhood as a child may be an omitted confounder). These are all plausible and rather modest suggestions if we look at the correlation matrix from Table 6 in Papageorge and Thom^19^. Following the derivations provided in the Supplementary Information, the inflation bias would be 0.021 under these assumptions, which explains a 16% downward change of the polygenic score coefficient. We provide detailed calculations for this case in the Supplementary Information.

We also show in Fig. 1B that the described bias results in greater values of explained variance statistics: these are R-squared values in the case of our simulations. This is because statistical models suffering from this bias explain both true and artificial (due to collider) variation in a dependent variable as we show in the derivations in the Supplementary Information. This further complicates the assessment of the relative predictive power of polygenic scores and environments. As demonstrated in the bottom panel of Fig. 1B, R-squared statistics for the model with an endogenous covariate and a polygenic score would include not only the true share of the variance in Y explained by G and E, but also the elements of variance that are due to rGE and confounder(s), U.

To conclude, the inclusion of associated polygenic scores and covariates in regression models may result in spurious estimates and greater explained variance statistics. The direction and strength of coefficient bias depend on the strength of the gene-covariate correlation and on the underlying structure of any endogeneity which links the covariate to the outcome variable.

### Gene × environment interaction models

A growing literature estimates the moderating patterns of environmental risks in the associations between polygenic scores and phenotypes. Here, in the same fashion as in additive models, environmental exposures of interest are not usually exogenous. For example, recent studies on gene-environment interaction analysis consider such environments as physical activity^34^, relationship status^35^, educational attainment^36^, lifestyle^37^, occupational exposure^38^, neighbourhood characteristics^39^ and others. There is an ongoing discussion on the implications of non-exogeneity of environments^7,40,41^. Also, the issue of collider bias has been demonstrated in the context of case-only gene-environment interaction studies^42^. We expand on these concerns by showing that moderation models also suffer from collider bias.

**Figure 2:**
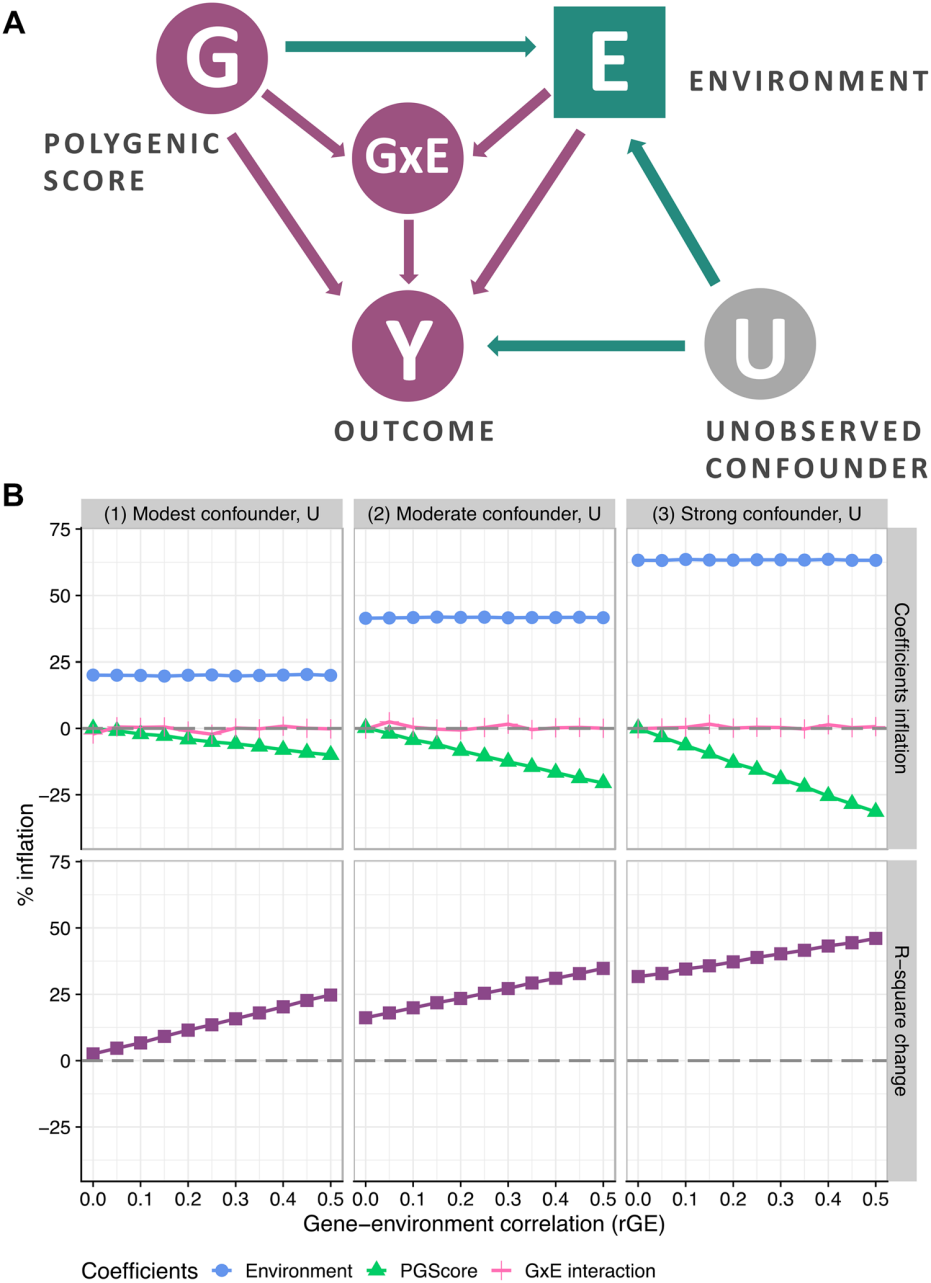
Collider bias in polygenic gene-environment interaction models. Panel A. Schematic diagram of the collider bias which occurs between polygenic score, environment, and outcome in cases of gene-environment interdependence. Dark purple circles represent variables, unobserved confounders are shown in grey circles, collider variables are indicated in squares. By adding E into the model with the polygenic score G, we make E a collider. A collider that is not conditioned on, blocks the path between its sources (G and U); once a collider is controlled for, the path is opened as indicated by green nodes. Panel B (top). Spurious regression estimates for the polygenic score and environment along with non-inflated interaction terms from the series of OLS simulations reflecting a range of gene-environment interdependence and the presence of modest, moderate, or strong confounder, U. Collider bias due to positive values of gene-environment correlation and the presence of an uncontrolled confounder, which is positively correlated with covariate and outcome, results in deflation of polygenic score estimates. Deflation is greater the higher the gene-environment correlation; greater confounding also results in greater bias. The interaction term is not affected but results for moderation analysis are biased as long as direct effects are spurious. Panel B (bottom). R-squared inflation plot from the series of OLS simulations; collider bias results in inflated values of explained variance statistics. R-squared statistics for the model with endogenous covariate and polygenic score includes not only the true share of the variance in Y explained by G and E (baseline estimate indicated by 0), but also the elements of variance that are due to gene-environment correlation and confounder(s), U.

Firstly, the problem outlined until this point is also relevant for gene-environment interaction studies. One difference, however, between additive and moderation models is the presence of the G × E interaction in the set of covariates. As indicated in Fig. 2A by green nodes, the bias path from G to Y through E and U would still lead to spurious results. Considering the examples of G × E studies mentioned earlier, the environments may, to some degree, be products of self-selection, which leads to a greater likelihood of G and E interdependence along with the presence of unobserved confounder(s), U. Secondly, since the overall G × E interaction pattern depends on the direct estimates of G on Y and E on Y, results for moderation analyses are biased when direct effects are spurious. However, the G × E coefficient per se is not inflated due to collider bias. This can be seen in Fig. 2B, along with the inflation of R-square statistics which were obtained from similar simulations as earlier but with the inclusion of interaction terms. Our insights are in line with Bun and Harrison^43^ who provide mathematical annotations and show that OLS estimation of endogenous interaction terms is consistent. The authors also highlight that this consistency applies only to interaction coefficients and not to the overall marginal effect.

The inflation of the GxE interaction term is not observed under the scenario where confounder, U, does not interact with a covariate, E, and polygenic score, G. This setting is considered in Bun and Harrison^43^ and in our simulation analyses presented in Fig. 2. However, because of the non-experimental nature of GxE analyses, we might suspect moderation between an omitted confounder, U, and various covariates in the model. Under this scenario, the estimation of interaction terms would be spurious and the range of concerns raised by Keller^27^ would directly apply here. In line with our argument, it is important to highlight that if there is E×U interaction on the path between E and Y (and thus on the indirect path linking G and Y), our estimate of the GxE term would be spurious due to collider bias. We provide an illustration of such a case in Figure S1 where the simulations show that G×E coefficients are biased in proportion to the strength of rGE and the unobserved confounder, U. Consequently, endogeneity of environmental covariates biases both additive and moderation models.

## Solutions

To address this issue, it is important to understand the nature of the correlation between polygenic scores and environment (or phenotype if it is used as a covariate)—whether a correlation is conditional and observed because of omitted confounders between G and E and/or a correlation reflects causal dependency. The former would necessitate controlling for the confounding factor: this could be parental characteristics (passive rGE)^23,44^, ancestry^2^, and so forth. If a correlation arises as a result of pleiotropy, active or evocative selection, the assumption of non-causal association would be violated and require another set of solutions to avoid collider bias. The latter is relevant when a phenotypic variable is used as a covariate along with its polygenic prediction since the association would be at least partially causal.

To obtain unbiased estimates, we need to apply causal inference methods that seek to exploit the exogenous variation in an environmental covariate. A comprehensive discussion of methods available for researchers and applicable to the context of this paper is provided by Fletcher and Conley^6^. Briefly, techniques such as regression discontinuity and difference-in-difference designs, instrumental variables and quasi-natural experiments will produce unbiased results for both additive and gene-environment interaction models, conditional on certain assumptions being met. As an illustrative case, we considered the instrumental variable solution and include it in our code, which is available online. However, any technique that would disentangle the exogenous variation in an environmental covariate would produce estimates which are not distorted by collider bias; hence, a choice of a particular model would depend on the case in question and a set of assumptions that are unique for each method.

There are also existing ways to assess the magnitude of the bias for the general collider cases^45,46^. Since the type of collider we described here is the product of both observed and unobserved factors, calculation of bias magnitude would rely on additional assumptions about the structure of the error correlation between the environmental covariate and the outcome. It is possible to assess the strength of gene-covariate association by either directly measuring their correlation in the data or by taking into account genetic correlations for the trait in question. The influence of unobserved variables on the bias, however, makes it impossible to provide a definitive estimate of the issue. In the Supplementary Information, we show the sources of bias in coefficient estimates and error in R-squared. In line with the results of our simulation analyses, mathematical expressions also demonstrate that the strength of collider bias is positively associated with the strength of rGE and unobserved confounders. The use of sensitivity analyses is a valuable tool in showing how robust conclusions are to different degrees of unobserved confounding and, thus, of collider bias^47^.

## Conclusion

We have discussed methodological considerations arising due to heritable environments (or phenotypes that are included into models as covariates) that have not yet been previously recognised. We demonstrated that the inclusion of environments that are products of polygenic scores may introduce endogenous selection bias through conditioning on a collider, leading to spurious associations. Particularly, we showed that the degree of bias depends on the strength of the gene-covariate correlation and of the omitted variable(s) linking the covariate and outcome. We also showed that the portion of explained variance is overestimated proportionally. We proposed some solutions that exploit the strength of causal inference methods: these are likely to be important not only for obtaining reliable results but also in the interpretation of existing studies.

## Methods

### Graphical models

We use a graphical approach to demonstrate the methodological problems. For the theory behind graphical models, see Pearl et al.^48^ The graphical approach is a transparent method to demonstrate the biases and it does not require any parametric assumptions which is an advantage. We also provide equations and detailed derivations for the bias under the assumptions of linear regression in Supplementary Information to compliment graphical models. In the schematic diagrams (or directed acyclic graphs) presented in the paper, arrows indicate associated paths, dark purple circles represent observed variables, grey circles represent unobserved variables (which are confounders in our case), and collider variables are indicated by squares.

### Simulations

To further illustrate this bias, we conducted a series of simulations of the simple linear models. All simulations are detailed in Supplementary Information. All analyses code is publicly available at: https://github.com/eva-akimova/collider-simulations.git (https://doi.org/10.5281/zenodo.4184672), to reproduce the figures presented in the paper. Simulations were conducted in R^49^ using dplyr^50^, broom^51^, purrr^52^, mvtnorm^53^, ggplot2^54^, cowplot^55^, tidyr^56^, AER^57^, forcats^58^ packages. First, we simulated scenarios of OLS regression for additive models. We allowed the G-E correlation to vary from 0 indicating no heritability to 0.5 indicating a highly heritable covariate. We considered three scenarios where U is a modest, moderate, or strong confounder of the E-Y association (r = 0.12, r = 0.25, and r = 0.38 respectively). We also considered the presence of direct effects from G to Y and E to Y which coefficients are both positive and 0.6. Second, for the gene-environment interaction models, we simulated the same settings and added the GxE coefficient at a fixed value of 0.1 for all scenarios.

## Supplementary information

Supplementary Information includes the detailed derivations for the bias under the assumption of linear relationships that are modelled using regression analysis and detailed information on simulations.

## Supplementary Information

Supplementary Information.

## Data Availability

The code for the simulations and figures is available on Zenodo (https://doi.org/10.5281/zenodo.4184672) and GitHub (https://github.com/eva-akimova/collider-simulations.git).

## References

[1] Barbaro N, Boutwell BB, Barnes JC, Shackelford TK (2017). Genetic confounding of the relationship between father absence and age at menarche. Evol. Hum. Behav..

[2] Mostafavi H, Harpak A, Agarwal I, Conley D, Pritchard JK, Przeworski M (2020). Variable prediction accuracy of polygenic scores within an ancestry group. Elife.

[3] Ware EB, Schmitz LL, Faul J, Gard A, Mitchell C, Smith JA, Zhao W, Weir D, Kardia SL (2017). Heterogeneity in polygenic scores for common human traits. BioRxiv.

[4] Morris TT, Davies NM, Hemani G, Smith GD (2020). Population phenomena inflate genetic associations of complex social traits. Sci. Adv..

[5] Conley D (2009). The promise and challenges of incorporating genetic data into longitudinal social science surveys and research. Biodemography Soc. Biol..

[6] Fletcher JM, Conley D (2013). The challenge of causal inference in gene-environment interaction research: leveraging research designs from the social sciences. Am. J. Public Health.

[7] Dudbridge F, Fletcher O (2014). Gene-environment dependence creates spurious gene-environment interaction. Am. J. Human Genet..

[8] Avinun R (2019). The E is in the G: gene–environment–trait correlations and findings from Genome-Wide Association Studies. Perspect. Psychol. Sci..

[9] Ni G, van der Werf J, Zhou X, Hyppönen E, Wray NR, Lee SH (2019). Genotype–covariate correlation and interaction disentangled by a whole-genome multivariate reaction norm model. Nat. Commun..

[10] Munafò MR, Tilling K, Taylor AE, Evans DM, Davey Smith G (2017). Collider scope: when selection bias can substantially influence observed associations. Int. J. Epidemiol..

[11] Conley D, Zhang S (2018). The promise of genes for understanding cause and effect. Proc. Natl. Acad. Sci..

[12] Plomin R, DeFries JC, Loehlin JC (1977). Genotype-environment interaction and correlation in the analysis of human behavior. Psychol. Bull..

[13] Infante-Rivard C, Cusson A (2018). Reflection on modern methods: selection bias—a review of recent developments. Int. J. Epidemiol..

[14] Day FR, Loh P-R, Scott RA, Ong KK, Perry JRB (2016). A robust example of collider bias in a genetic association study. Am. J. Human Genet..

[15] Stergiakouli E, Martin J, Hamshere ML, Heron J, St Pourcain B, Timpson NJ, Thapar A, Davey Smith G (2016). Association between polygenic risk scores for attention-deficit hyperactivity disorder and educational and cognitive outcomes in the general population. Int. J. Epidemiol..

[16] Rutten-Jacobs LC, Larsson SC, Malik R, Rannikmäe K, Sudlow CL, Dichgans M, Markus HS, Traylor M (2018). Genetic risk, incident stroke, and the benefits of adhering to a healthy lifestyle: cohort study of 306 473 UK Biobank participants. BMJ.

[17] Yun, J.-S., Jung, S.-H., Shivakumar, M., Xiao, B., Khera, A. V., Park, W.-Y., Won, H.-H. & Kim, D. Polygenic risk, lifestyle, and cardiovascular mortality: a prospective population-based UK Biobank study. medRxiv, (2021).

[18] Ayorech Z, Plomin R, von Stumm S (2019). Using DNA to predict educational trajectories in early adulthood. Dev. Psychol..

[19] Papageorge NW, Thom K (2019). Genes, education, and labor market outcomes: evidence from the health and retirement study. J. Eur. Econ. Assoc..

[20] Smith-Woolley E, Pingault J-B, Selzam S, Rimfeld K, Krapohl E, von Stumm S, Asbury K, Dale PS, Young T, Allen R (2018). Differences in exam performance between pupils attending selective and non-selective schools mirror the genetic differences between them. npj Sci. Learn..

[21] Jaffee SR, Price TS (2007). Gene–environment correlations: a review of the evidence and implications for prevention of mental illness. Mol. Psychiatry.

[22] Mills MC, Barban N, Tropf FC (2020). An Introduction to Statistical Genetic Data Analysis.

[23] Kong A, Thorleifsson G, Frigge ML, Vilhjalmsson BJ, Young AI, Thorgeirsson TE, Benonisdottir S, Oddsson A, Halldorsson BV, Masson G (2018). The nature of nurture: effects of parental genotypes. Science.

[24] Avinun R, Knafo A (2014). Parenting as a reaction evoked by children’s genotype: a meta-analysis of children-as-twins studies. Pers. Soc. Psychol. Rev..

[25] Klahr AM, Burt SA (2014). Elucidating the etiology of individual differences in parenting: a meta-analysis of behavioral genetic research. Psychol. Bull..

[26] van Rheenen W, Peyrot WJ, Schork AJ, Lee SH, Wray NR (2019). Genetic correlations of polygenic disease traits: from theory to practice. Nat. Rev. Genet..

[27] Bulik-Sullivan B, Finucane HK, Anttila V, Gusev A, Day FR, Loh P-R, Duncan L, Perry JRB, Patterson N, Robinson EB (2015). An atlas of genetic correlations across human diseases and traits. Nat. Genet..

[28] Abbott, L., Bloom, J., Bryant, S., Carey, C., Churchhouse, C., Ganna, A., Goldstein, J., Howrigan, D., King, D., Neale, B. *et al*. Genetic correlation between traits and disorders in the UK Biobank, (2020). https://ukbb-rg.hail.is

[29] Kerminen S, Martin AR, Koskela J, Ruotsalainen SE, Havulinna AS, Surakka I, Palotie A, Perola M, Salomaa V, Daly MJ (2019). Geographic variation and bias in the polygenic scores of complex diseases and traits in Finland. Am. J. Human Genet..

[30] Elwert F, Winship C (2014). Endogenous selection bias: the problem of conditioning on a collider variable. Annu. Rev. Sociol..

[31] Lindsay S, Jennie Jacobsk R (2009). The influence of childhood poverty on the self-management of heart disease in later life. Social Sources of Disparities in Health and Health Care and Linkages to Policy, Population Concerns and Providers of Care.

[32] Lawlor DA, Davey Smith G, Patel R, Ebrahim S (2005). Life-Course Socioeconomic position, area deprivation, and coronary heart disease: findings from the british women’s heart and health study. Am. J. Public Health.

[33] Kromhout D, Menotti A, Kesteloot H, Sans S (2002). Prevention of coronary heart disease by diet and lifestyle. Circulation.

[34] Wu, Y. Y., Thompson, M. D., Youkhana, F. & Pirkle, C. M. Interaction between physical activity and polygenic score on type 2 diabetes mellitus in older black and white participants from the health and retirement study. *J. Gerontol. Ser. A*, (2021). 10.1093/gerona/glab025 PMC8355465 33515027

[35] Barr PB, Kuo SI-C, Aliev F, Latvala A, Viken R, Rose RJ, Kaprio J, Salvatore JE, Dick DM (2019). Polygenic risk for alcohol misuse is moderated by romantic partnerships. Addiction.

[36] Amin V, Böckerman P, Viinikainen J, Smart MC, Bao Y, Kumari M, Pitkänen N, Lehtimäki T, Raitakari O, Pehkonen J (2017). Gene-environment interactions between education and body mass: evidence from the UK and Finland. Soc. Sci. Med..

[37] Ye Y, Chen X, Han J, Jiang W, Natarajan P, Zhao H (2021). Interactions between enhanced polygenic risk scores and lifestyle for cardiovascular disease, diabetes, and lipid levels. Circ. Genomic Precis. Med..

[38] Zeng X, Vonk JM, van der Plaat DA, Faiz A, Paré PD, Joubert P, Nickle D, Brandsma C-A, Kromhout H, Vermeulen R (2019). Genome-wide interaction study of gene-by-occupational exposures on respiratory symptoms. Environ. Int..

[39] Robinette JW, Boardman JD, Crimmins EM (2019). Differential vulnerability to neighbourhood disorder: a gene×environment interaction study. J. Epidemiol. Commun. Health.

[40] Schmitz L, Conley D (2017). Modeling gene-environment interactions with quasi-natural experiments. J. Pers..

[41] Keller MC (2014). Gene × environment interaction studies have not properly controlled for potential confounders: the problem and the (simple) solution. Biol. Psychiatry.

[42] Balazard F, Le Fur S, Bougnères P, Valleron A-J (2017). Interactions and collider bias in case-only gene-environment data. BioRxiv.

[43] Bun MJG, Harrison TD (2019). OLS and IV estimation of regression models including endogenous interaction terms. Economet. Rev..

[44] Trejo S, Domingue BW (2018). Genetic nature or genetic nurture? Introducing social genetic parameters to quantify bias in polygenic score analyses. Biodemography Soc. Biol..

[45] VanderWeele TJ (2010). Bias formulas for sensitivity analysis for direct and indirect effects. Epidemiology.

[46] Greenland S (2003). Quantifying biases in causal models: classical confounding vs collider-stratification bias. Epidemiology.

[47] Ding P, VanderWeele TJ (2016). Sensitivity analysis without assumptions. Epidemiology.

[48] Pearl J, Glymour M, Jewell NP (2016). Causal Inference in Statistics: A Primer.

[49] Team, R. C. R: A language and environment for statistical computing, (2013).

[50] Wickham, H., François, R., Henry, L. & Müller, K. dplyr: A Grammar of Data Manipulation. R package version 1.0.5, (2021).

[51] Robinson, D., Hayes, A., & Couch, S. broom: Convert Statistical Objects into Tidy Tibbles. R package version 0.7.2, (2020).

[52] Henry, L. & Wickham, H. purrr: Functional Programming Tools. R package version 0.3.4, (2020).

[53] Genz, A., Bretz, F., Miwa, T., Mi, X., Leisch, F., Scheipl, F. & Hothorn, T. mvtnorm: Multivariate Normal and t Distributions. R package version 1.1-1, (2020).

[54] Wickham H (2016). ggplot2: Elegant Graphics for Data Analysis.

[55] Wilke, C. O. cowplot: Streamlined Plot Theme and Plot Annotations for 'ggplot2'. R package version 1.1.0, (2020).

[56] Wickham, H. tidyr: Tidy Messy Data. R package version 1.1.2, (2020).

[57] Kleiber C, Zeileis A (2008). Applied Econometrics with R.

[58] Wickham, H. forcats: Tools for Working with Categorical Variables (Factors). R package version 0.5.0, (2020).

