# Supplementary material for: Gene-environment dependencies lead to collider bias in models with polygenic scores
Source: Sci Rep. 2021 May 4;11:9457. doi: 10.1038/s41598-021-89020-x (PMC8097011; doi:10.1038/s41598-021-89020-x)
Supplement: Supplementary file 1 — Supplementary Information. [file 41598_2021_89020_MOESM1_ESM.pdf]

Supplementary Information

Gene-environment dependencies lead to collider bias in models with polygenic scores

Evelina T. Akimova,[1,2,*] Richard Breen, [1,3] David M. Brazel,[2,3] Melinda C. Mills[2,3]

[1] Department of Sociology, University of Oxford, Oxford, OX1 1JD, United Kingdom
[2] Leverhulme Centre for Demographic Science, University of Oxford, Oxford, OX1 1JD, United Kingdom
[3] Nuffield College, University of Oxford, Oxford, OX1 1NF, United Kingdom

*Corresponding author. Email address: evelina.akimova@sociology.ox.ac.uk

S1. Expressions for the bias in gene-environment models due to interdependency of polygenic scores and environments

The main text provided a general description of endogenous selection bias when polygenic scores and environments are not independent. Here we further illustrate this issue by deriving exact expressions for the bias under the assumption of linear relationships that can be modelled using regression analysis.

We assume that the data have been generated by the DAG shown in Figure 1A. Here U is an unobserved variable or set of variables that confounds the E – Y relationship (this is equivalent to, but, in our view more transparent than, a depiction that would include correlated error terms for E and Y). We further assume that all the variables have unit standard deviation and that G is exogenous.

S1.1 Additive model

When the effects of G and E on Y are additive the true linear models given the data-generating process are:

$$E(E|G,U) = \alpha_0 + \alpha_1 G + \alpha_2 U \qquad \text{(Equation S1)}$$

$$E(Y|G,E,U) = \beta_0 + \beta_1 G + \beta_2 E + \beta_3 U \qquad \text{(Equation S2)}$$

We assume that the parameters α_1, β_1, and β_2 are all positive.

If estimate the models:

$$E(E|G) = a_0 + a_1 G \qquad \text{(Equation S3)}$$

$$E(Y|G,E) = b_0 + b_1 G + b_2 E \qquad \text{(Equation S4)}$$

the relationships between the true and estimated parameters are:

$$b_1 = \beta_1 - \frac{\alpha_1 \alpha_2 \beta_3}{1-\alpha_1^2} \qquad \text{(Equation S5)}$$

$$b_2 = \beta_2 + \frac{\alpha_2 \beta_3}{1-\alpha_1^2} \qquad \text{(Equation S6)}$$

The proof is as follows. Let β_{YG} denote the coefficient of the unconditional regression of Y on G and likewise for β_{YE}. Then, tracing the paths linking G and Y in the Figure A1a we have:

$$\beta_{YG} = \beta_1 + \beta_2 \alpha_1$$

and

$$\beta_{YE} = \beta_2 + \beta_1 \alpha_1 + \beta_3 \alpha_2$$

Given that $\beta_{EG} = \alpha_1$ we then apply the standard formula to derive conditional regression coefficients from unconditional:

$$b_1 = \frac{\beta_{YG} - \beta_{YE}\alpha_1}{1-\alpha_1^2} = \frac{\beta_1 + \beta_2\alpha_1 - (\beta_2 + \beta_1\alpha_1 + \beta_3\alpha_2)\alpha_1}{1-\alpha_1^2}$$

Straightforward algebra yields (S5). b_2 is derived similarly. Notice, however, that $a_1 = \alpha_1$ because G and U are unconditionally independent.

The bias in both estimates depends on the sign of $\alpha_2 \times \beta_3$: if this is positive the estimate of the partial effect of G on Y, given E, will be downwardly biased and the estimate of the effect of E on Y, given G, will be upwardly biased. If there is no correlation between G and E ($\alpha_1 = 0$) then $b_1$will be unbiased. If there is no

effect of an unmeasured confounder (either $\alpha_2 = 0$ and/or $\beta_3 = 0$) both b_1 and b_2 will be unbiased. The bias in the effect of G on Y has a different sign than the bias in the effect of E on Y: if the bias in the latter is positive, the size of the genetic effect will be underestimated relative to the environmental effect.

The example of coefficient deflation from Papageorge and Thom[1] can be demonstrated following Equation S5. For instance, considering the case on nonroutine interactive job tasks as the dependent variable, we see that the baseline coefficient of the educational attainment polygenic score is 0.185, which reflects a model without any environmental and phenotypic covariates (Table 6 in Papageorge and Thom[1]). In the model with educational controls (respondent's years of schooling and parental education), the polygenic score coefficient drops to 0.055 reflecting a 70% negative change. Since the dependent variable is standardised, we can assess the relative importance of collider bias which is $\frac{\alpha_1\alpha_2\beta_3}{1-\alpha_1^2}$ from Equation S5 under additional assumptions. If we allow the coefficient of the correlation between educational attainment polygenic score and respondents years of schooling α_1=0.300, and the presence of unobserved confounder U, positively correlated with both years of schooling and job task (for example, living in advantaged neighbourhood as a child), we have $\alpha_2 = 0.250$ and $\beta_3 = 0.250$. These are all plausible and rather modest suggestions following correlation matrix from Table 6 in Papageorge and Thom[1], leading the inflation bias to be:

$$\frac{\alpha_1\alpha_2\beta_3}{1-\alpha_1^2} = \frac{0.300 \times 0.250 \times 0.250}{1-0.300^2} = 0.021$$

which explains 16% downward change of polygenic score coefficient.

S1.2 G×E interaction model

The DAG in Figure 2A shows the case in which the effect of G on Y varies with E. In this case, the true linear models given the data-generating process are:

$$E(E|G,U) = \alpha_0 + \alpha_1 G + \alpha_2 U \quad \text{(Equation S7)}$$

$$E(Y|G,E,U) = \beta_0 + \beta_1 G + \beta_2 E + \beta_3 U + \beta_4 (GE) \quad \text{(Equation S8)}$$

We estimate:

$$E(E|G) = a_0 + a_1 G \quad \text{(Equation S9)}$$

$$E(Y|G,E) = b_0 + b_1 G + b_2 E + b_4 GE \quad \text{(Equation S10)}$$

In this case, b_4 is an unbiased estimate of β_4 because the backdoor path from G-E to Y is blocked by E. The bias in b_1 and b_2 will be the same as above. In the case in which E is a binary variable, coded 0 and 1, b_4 will be an unbiased estimate of the difference in the effect of G at $E = 1$ and $E = 0$, but the estimate of the baseline effect of G on Y when $E = 0$ will be biased.

S1.3 Bias in R^2

The R^2 for models S4 and S10 will be biased. In the additive case, for example, the true R^2 attributable to G and E is:

$$\frac{\beta_1^2 var(G) + \beta_2^2 var(E) + 2\beta_1\beta_2 cov(G,E)}{var(Y)} = \beta_1^2 + \beta_2^2 + 2\beta_1\beta_2\alpha_1 \quad \text{(Equation S11)}$$

(using the assumption that all the variables have unit standard deviation). But the reported R^2 from model S4 is:

$$b_1^2 + b_2^2 + 2b_1b_2\alpha_1 \qquad \text{(Equation S12)}$$

Substituting S5 and S6 into S12 we calculate the inflation of R^2 due to confounding and collider bias. This is:

$$R^2 bias = \alpha_2\beta_3\left[\frac{\alpha_2\beta_3}{1-\alpha_1^2} + 2\beta_2\right] \qquad \text{(Equation S13)}$$

Confounder bias arises from $\alpha_2\beta_3$. The derivative of S13 with respect to this is positive provided that $1 - \alpha_1^2 > 0$. The derivative of S13 with respect to α_1 (which captures the association between G and E) is:

$$\frac{2\alpha_1\alpha_2^2\beta_3^2}{(1-\alpha_1^2)^2}$$

The sign of this depends on the sign of the numerator. When it is positive both confounding and collider bias will inflate the reported R^2. As an example, consider a case in which $\beta_1 = 0.465, \beta_2 = 0.505, \beta_3 = 0.231, \alpha_1 = 0.209, \alpha_2 = 0.693$. Then the observed $R^2 = 0.758$, whereas the true share of the variance in Y explained by G and E is 0.569. The inflation bias here is:

$$0.693 \times 0.231\left[\frac{0.693 \times 0.231}{1 - 0.209^2} + 2 \times 0.505\right] = 0.188$$

If the correlation between G and E had been larger and/or if the confounding of E had been greater, the reported R^2 would have been larger because of the greater bias.

S2. Simulation analyses

The code for the simulations and figures is available on Zenodo (DOI: 10.5281/zenodo.4184673) and GitHub (https://github.com/eva-akimova/collider-simulations.git). For the figures presented in the main text, we simulated scenarios of OLS regressions where G – E association varies between 0 and .5; G – Y and E – Y coefficients are both positive and .6; uncontrolled confounder, U, is positively and modestly, moderately or strongly correlated with covariate, E, and outcome, Y, (r = .12, r = .25, and r = .38 respectively for the three scenarios). For the gene-environment interaction models we simulated the same settings and added GxE coefficient at a fixed value of 0.1 for all scenarios.

Here, we expand our simulation analyses and further illustrate the bias in gene-environment interaction models where unobserved confounder, U, interacts with covariate, E, at a fixed value of 0.2 for all scenarios. Simulation results presented below in Figure S1 along with a DAG to illustrate the bias.

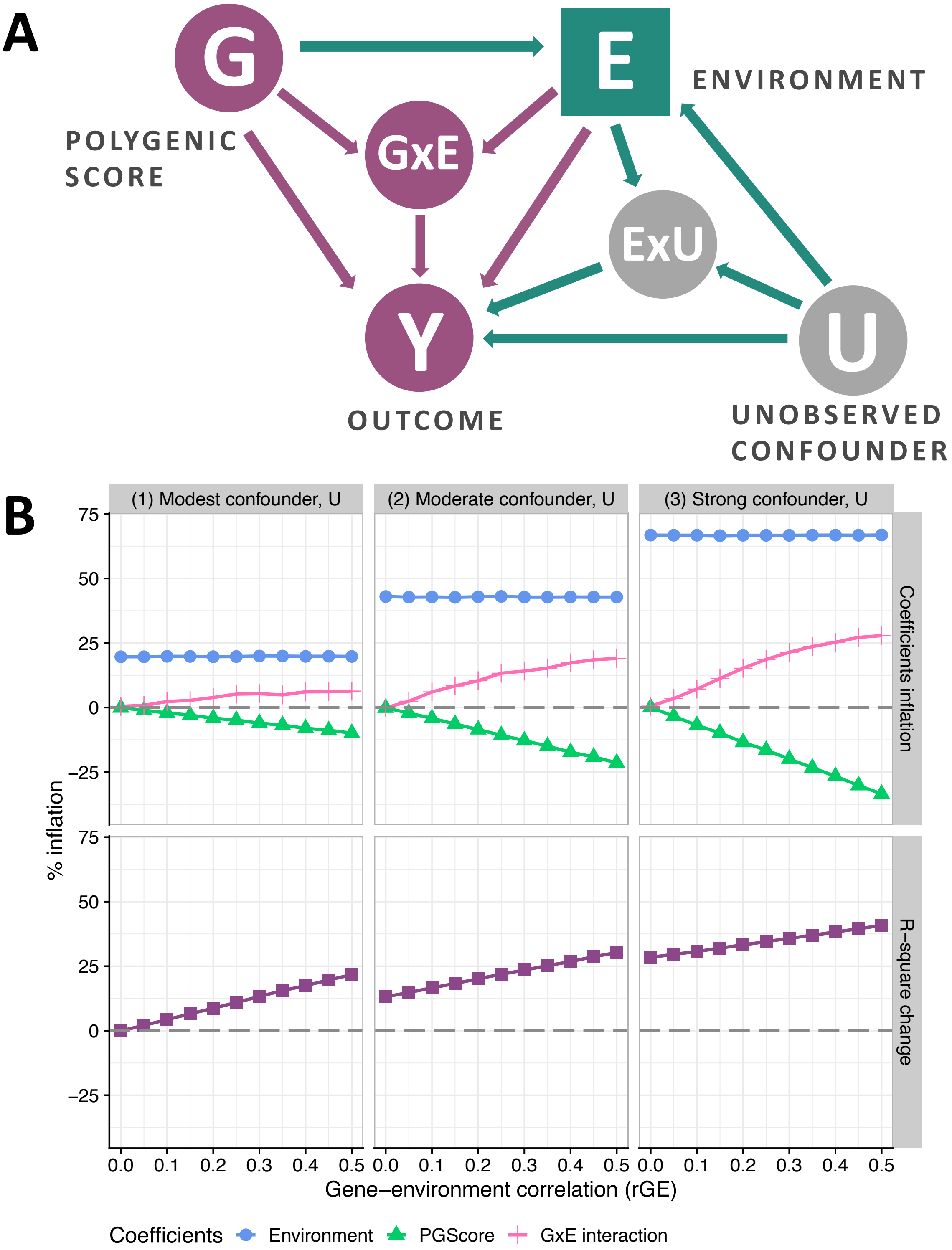

Figure S1. Collider bias in polygenic gene-environment interaction models

Figure S1. Collider bias in polygenic gene-environment interaction models. Panel A. Schematic diagram of the collider bias which occurs between polygenic score, environment, and outcome in cases of gene-environment interdependence. Dark purple circles represent variables, unobserved confounders and it interaction term with E are shown in grey circles, collider variables are indicated in squares. By adding E into the model with the polygenic score G, we make E a collider. A collider that is not conditioned on, blocks the path between its sources (G and U); once a collider is controlled for, the path is opened as indicated by green nodes. ExU interaction term is also on the bias path once E is conditioned on. Panel B (top). Spurious regression estimates for the polygenic score and environment along with inflated interaction terms from the series of OLS simulations reflecting a range of gene-environment interdependence and the presence of modest, moderate, or strong confounder, U. Collider bias due to positive values of gene-environment correlation and the presence of an uncontrolled confounder, which is positively correlated with covariate and outcome, results in deflation of polygenic score estimates. Deflation is greater the higher the gene-environment correlation; greater confounding also results in greater bias. The interaction term is affected proportionally to the strength of rGE and unobserved confounder, U. Panel B (bottom). R-squared inflation plot from the series of OLS simulations; collider bias results in inflated values of explained variance statistics. R-squared statistics for the model with endogenous covariate and polygenic score includes not only the true share of the variance in Y explained by G and E (baseline estimate indicated by 0), but also the elements of variance that are due to gene-environment correlation and confounder(s), U.

References

1. Papageorge, N.W., and Thom, K. (2019). Genes, education, and labor market outcomes: Evidence from the Health and Retirement Study. Journal of the European Economic Association jvz072.
